# OPERA: a phase II study of DHP107 (oral paclitaxel) versus intravenous paclitaxel in patients with HER2-negative recurrent or metastatic breast cancer

**DOI:** 10.1007/s10549-026-07944-2

**Published:** 2026-03-30

**Authors:** Hope S. Rugo, T. J. Pluard, P. Sharma, M. Melisko, G. Al-Jazayrly, Y. Ji, N. Vidula, J. Ellerton, M. Smakal, M. Zimovjanova, D. Weng

**Affiliations:** 1 Breast Medical Oncology, Department of Medical Oncology & Therapeutics Research, City of Hope Comprehensive Cancer Center, Women’s Cancers Program, Duarte, CA USA; 2 Medical Oncology Department, Saint Luke’s Hospital of Kansas City—Saint Luke’s Health System, Kansas City, KS USA; 3 Internal Medicine Department, University of Kansas Medical Center, Westwood, KS USA; 4 University of California San Francisco Comprehensive Cancer Center, San Francisco, CA USA; 5 Oncology Department, HPMC—Hollywood Presbyterian Medical Center, Los Angeles, CA USA; 6 Hem/Onc Department, Health Partners Regions Hospital, St. Paul, Minneapolis, MN USA; 7 Hematology/Medical Oncology Department, Massachusetts General Hospital, Boston, MA USA; 8 Nevada NCORP, Las Vegas, NV USA; 9 Oncology Department, Hospital Hořovice, Hořovice, Czech Republic; 10 Oncology Department, General Teaching Hospital and The First Faculty of Medicine of Charles University in Prague, Prague, Czech Republic; 11 Oncology & Hematology, Anne Arundel Medical Center, Annapolis, USA

**Keywords:** DHP107 (oral paclitaxel), HER2-negative, Phase II, Recurrent or metastatic breast cancer

## Abstract

**Purpose:**

DHP107 is an oral paclitaxel enabling administration of paclitaxel without Cremophor EL, a vehicle used to improve the solubility of intravenous (IV) paclitaxel. The randomized phase II OPERA study investigated the efficacy and safety of DHP107 versus IV paclitaxel in patients with HER2-negative breast cancer.

**Methods:**

OPERA was conducted in the USA and Czech Republic. Patients were ≥ 18 years, with measurable disease, and histologically or cytologically confirmed recurrent or metastatic breast cancer with any tumor hormone receptor status. Patients were randomized 2:1 to DHP107 (200 mg/m^2^ po bid with premedication if needed on days 1, 8, and 15, every 28 days) or IV paclitaxel (80 mg/m^2^ with standard premedication on days 1, 8, and 15 every 28 days). The primary objective was DHP107 efficacy; secondary objectives included DHP107 safety and tolerability.

**Results:**

72 patients were randomized, 48 to DHP107 and 24 to IV paclitaxel. There was one complete response and 11 partial responses with DHP107 (objective response rate [ORR 25.0%; 90% CI 15.1–37.3), and six partial responses with IV paclitaxel (objective response rate [ORR] 28.6%; 90% CI 13.2–48.7; *p* = 0.7559). Median progression-free survival (PFS) was 5.5 months for DHP107 and 4.7 months for IV paclitaxel (*p* = 0.8018); median overall survival (OS) was 17.1 and 13.2 months, respectively (*p* = 0.7629). Common all-grade adverse events were diarrhea (68.8%), nausea (64.6%), and fatigue (52.1%) for DHP107 and fatigue (47.6%), peripheral neuropathy (42.9%), and alopecia (42.9%) for IV paclitaxel.

**Conclusion:**

DHP107 is a tolerable and feasible treatment for patients with recurrent or metastatic HER2-negative breast cancer, with similar efficacy and safety to IV paclitaxel.

**Clinicaltrials.gov no:** NCT03326102; date of registration October 19, 2017.

**Supplementary Information:**

The online version contains supplementary material available at 10.1007/s10549-026-07944-2.

## Introduction

Paclitaxel is an antimitotic agent with demonstrated antineoplastic effect in various malignancies including breast cancer [1]. Its use can be limited by associated adverse events (AEs), in particular peripheral neuropathy, hypersensitivity reactions, and bone-marrow suppression [2, 3]. Furthermore, poor water solubility necessitates the use of Cremophor EL (BASF Corporation, Ludwigshafen, Germany) as a vehicle to aid intravenous (IV) administration of paclitaxel, although its use is also associated with hypersensitivity reactions [4]. Considerable effort has, therefore, been invested in finding alternatives to IV administration of paclitaxel.

DHP107 is an oral paclitaxel created using a novel lipid formulation, the DaeHwa-Lipid bAsed Self-Emulsifying Drug delivery system (DH-LASED). This enables oral administration of paclitaxel without the need for Cremophor EL [5]. DHP107 was approved in the Republic of Korea in 2016 for the treatment of patients with advanced, metastatic, or locally recurrent gastric cancer, based on the results of the phase III DREAM study (NCT0183973) in which DHP107 showed comparable efficacy and safety versus IV paclitaxel, with no hypersensitivity reactions [6].

We now report findings from the randomized phase II OPERA study, which was designed to support the phase II and III OPTIMAL studies, results of which have been presented [7, 8]. OPERA examined the efficacy, safety, and pharmacokinetic (PK) profile of DHP107 versus IV paclitaxel in patients with recurrent or metastatic human epidermal growth factor receptor 2 (HER2)-negative breast cancer in the USA and the Czech Republic. Genetic variations between Asian and non-Asian patients, which lead to differences in tumor biology, treatment response, and drug metabolism, have been identified for several anticancer agents, including paclitaxel [9–11]. With this in mind, the OPERA study also aimed to examine the PK profile of DHP107 in non-Asian patients, enabling comparison of PK parameters after DHP107 administration with historical data for Asian patients.

## Methods

### Study design

OPERA was a multinational, multicenter, open-label, randomized, controlled phase II trial conducted at 15 sites in the USA and the Czech Republic (NCT03326102). The study was originally planned to confirm the feasibility of a phase III study and to support the approval by the US Food and Drugs Administration of DHP107 for the treatment of patients with breast cancer. As a result of the COVID-19 pandemic, enrollment of patients in the USA was not as high as expected and enrollment was expanded to include sites in the Czech Republic.

### Patients and treatment

Patients were ≥ 18 years of age, with measurable disease according to Response Evaluation Criteria In Solid Tumors (RECIST) version 1.1: histologically or cytologically confirmed recurrent or metastatic breast cancer (MBC) that was HER2-negative by immunohistochemistry or in situ hybridization assessment of samples from the primary tumor or metastases. Patients were eligible regardless of tumor hormone receptor status, i.e. with estrogen receptor (ER) or progesterone receptor (PR)-positive or -negative tumors. Patients had to have an Eastern Cooperative Oncology Group performance status of ≤ 2 and could have received up to three prior lines of systemic chemotherapy for advanced disease. Adequate organ function was required. Key exclusion criteria were prior taxane therapy in the metastatic setting, history of severe hypersensitivity reaction to the active ingredient or any excipients of DHP107 or IV paclitaxel, adjuvant or neoadjuvant treatment for early-stage breast cancer in the 6 months before study entry, and neuropathy grade ≥ 2 at the time of study entry.

Patients were randomly assigned in a 2:1 ratio to DHP107 or IV paclitaxel. DHP107 was administered orally twice daily (200 mg/m^2^) with premedication per investigator discretion, on days 1, 8, and 15 every 28 days. IV paclitaxel (80 mg/m^2^) was administered with standard premedication on days 1, 8, and 15 every 28 days. Treatment continued until evidence of disease progression, intolerable toxicity, or patient withdrawal.

### Outcomes and endpoints

The primary objective was to evaluate the efficacy of DHP107 in patients with recurrent or metastatic breast cancer. Secondary objectives were to evaluate the safety, tolerability, and PK profile of DHP107.

The primary efficacy endpoint was objective response rate (ORR), evaluated with RECIST version 1.1 every 8 weeks (± 7 days) from randomization. Secondary endpoints included progression-free survival (PFS), overall survival (OS), time to treatment failure (TTF), duration of response (DoR), and disease control rate (DCR; complete response + partial response + stable disease). Safety assessments included AEs according to National Cancer Institute Common Terminology Criteria for Adverse Events version 4.03, treatment-emergent AEs (TEAEs), and serious AEs.

PK endpoints included time to peak drug concentration (T_max_), drug half-life, maximum drug concentration (C_max_), area under the curve up to the last quantifiable time point (AUC_last_), and AUC from the first time point extrapolated to infinity for DHP107.

Health-related quality of life (HRQoL) was assessed using the European Organization for Research and Treatment of Cancer (EORTC) QLQ-C30 core quality of life questionnaire. This is designed to measure physical, psychological, and social function of people with cancer and is composed of multi-item scales and single items [12].

### Assessments

Tumor assessments were performed every two cycles (every 8 weeks ± 7 days) using computed tomography (CT) scans until disease progression or initiation of subsequent chemotherapy. For patients with hypersensitivity or documented history of hypersensitivity to CT contrast media, a magnetic resonance imaging scan could be performed at the investigator’s discretion.

PK analyses were performed in a subset of patients receiving DHP107. The PK study included all patients who received at least one dose of DHP107, had at least one post-dose PK measurement, and had no protocol deviations that would significantly affect the PK. For patients taking part in the PK study, blood samples were collected on day 1 of cycle 1 at pre-dose and 1, 2, 3, 4, 6, and 10 h post dose (before the second dose on day 1) and before the first dose on day 8 of cycle 1.

### Statistical analysis

As this was an exploratory study designed to evaluate the efficacy and safety of DHP107, and a hypothesis test was not required, a sample size of 72 patients with 10% attrition rate was considered sufficient to ensure that the lower one-sided 95% confidence limit for the true difference in response rates extended no more than 20% from the observed difference, assuming an observed ORR of 60% in both groups.

The following populations were analyzed: the full analysis set (FAS), which included all randomized patients who received DHP107 and for whom data were collected after randomization; the per-protocol set (PPS), which included all patients who received at least one cycle of treatment, had at least one tumor assessment, and had no major protocol violations; the safety set, which included all patients who had at least one dose of study drug; and the PK set.

The primary analysis compared DHP107 versus IV paclitaxel with respect to ORR using Pearson’s chi-square test or Fisher’s exact test. The ORR and DCR were reported as frequency, percentage, and 90% confidence interval (CI). PFS, OS, TTF, and DoR were evaluated using the Kaplan–Meier method. The unstratified log-rank test was used to compare the two treatment groups. For hazard ratios (HRs) of treatment groups, point estimates and 90% CI were obtained using unstratified and stratified Cox regressions with triple-negative breast cancer (TNBC) and disease-free interval (DFI) as stratification factors.

Statistical analyses were performed using SAS version 9.4 or higher (SAS Institute, Cary, NC, USA).

## Results

**Fig. 1:**
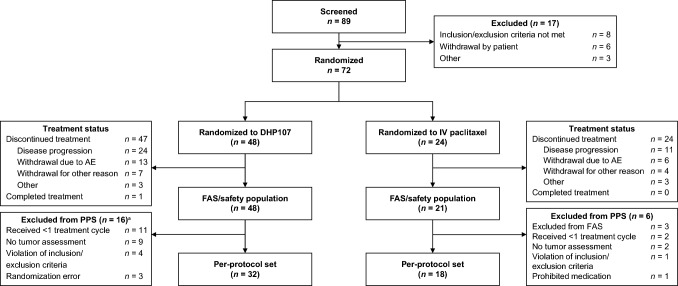
CONSORT diagram AE, adverse event; FAS, full analysis set; IV, intravenous; PPS, per-protocol set

Between July 2018 and June 2021, 72 patients were enrolled in the study; 48 were randomized to DHP107 and 24 were randomized to IV paclitaxel (Fig. 1). Three patients in the IV paclitaxel group were not treated; the FAS therefore consisted of 48 patients treated with DHP107 and 21 treated with IV paclitaxel. In total, 22 patients were excluded from the PPS (Fig. 1): 16 from the DHP107 group (11 received < 1 treatment cycle, nine had no tumor assessment, four had violations of inclusion/exclusion criteria, three had stratification errors that resulted in incorrect random numbers) and six from the IV paclitaxel group (two received < 1 treatment cycle; two had no tumor assessment; one had a violation of inclusion/exclusion criteria; three received no study drug; one received a prohibited concomitant medication–gosorelin acetate).

Patient characteristics at baseline are shown in Table 1. The median age was 60 years (range 36–84 years) and 56 patients (77.8%) were White. Common metastatic sites included bone (*n* = 55; 76.4%), liver (*n* = 45; 62.5%), and lymph nodes (*n* = 32; 44.4%). Twelve patients (16.7%) had TNBC and 24 patients (33.3%) had a DFI at baseline of ≤ 12 months. Fifty-eight patients (80.6%) had had prior chemotherapy, 14 (19.4%) in the neoadjuvant setting, 32 (44.4%) as adjuvant therapy, and 26 (36.1%) for metastatic disease.

**Table 1:** Patient characteristics at baseline (safety set; *n* = 72)

Characteristic	DHP107 (*n* = 48)	IV paclitaxel (*n* = 24)	Total (*n* = 72)
Median age, years (range)	60.5 (40–84)	58.5 (36–82)	60.0 (36–84)
Female sex, *n* (%)	48 (100)	24 (100)	72 (100)
Race, *n* (%)			
White	38 (79.2)	18 (75.0)	56 (77.8)
Asian	5 (10.4)	1 (4.2)	6 (8.3)
Black or African American	1 (2.1)	4 (16.7)	5 (6.9)
Other	4 (8.3)	1 (4.2)	5 (6.9)
ECOG performance status, *n* (%)			
0	19 (39.6)	7 (29.2)	26 (36.1)
1	27 (56.3)	12 (50.0)	39 (54.2)
2	2 (4.2)	2 (8.3)	4 (5.6)
Missing	0	3 (12.5)	3 (4.2)
Hormone receptor status, *n* (%)^a^			
Positive	40 (83.3)	20 (83.3)	60 (83.3)
Negative	8 (16.7)	4 (16.7)	12 (16.7)
Metastases at baseline, *n* (%)	47 (97.9)	22 (91.7)	69 (95.8)
Metastatic sites, *n* (%)			
Bone	36 (75.0)	19 (79.2)	55 (76.4)
Liver	31 (64.6)	14 (58.3)	45 (62.5)
Lymph node	23 (47.9)	9 (37.5)	32 (44.4)
Lung	15 (31.3)	8 (33.3)	23 (31.9)
Other	11 (22.9)	7 (29.2)	18 (25.0)
Missing	1 (2.1)	2 (8.3)	3 (4.2)
Disease-free interval ≤ 12 months, *n* (%)	16 (33.3)	8 (33.3)	24 (33.3)
Prior chemotherapy, *n* (%)	36 (75.0)	22 (91.7)	58 (80.6)
Anthracyclines	20 (41.7)	14 (58.3)	34 (47.2)
Cyclophosphamide	25 (52.1)	12 (50.0)	37 (51.4)
Pyrimidine analogs	32 (66.7)	16 (66.7)	48 (66.7)
Taxanes	25 (52.1)	11 (45.8)	36 (50.0)
Prior neoadjuvant chemotherapy, *n* (%)	8 (16.7)	6 (25.0)	14 (19.4)
Prior adjuvant chemotherapy, *n* (%)	24 (50.0)	8 (33.3)	32 (44.4)
Prior palliative chemotherapy, *n* (%)	14 (29.2)	12 (50.0)	26 (36.1)
Line 1	9 (18.8)	7 (29.2)	16 (22.2)
Line 2	4 (8.3)	4 (16.7)	8 (11.1)
Line 3	1 (2.1)	1 (4.2)	2 (2.8)
Prior endocrine therapy, *n* (%)	39 (81.3)	20 (83.3)	59 (81.9)
Prior surgery, *n* (%)	37 (77.1)	20 (83.3)	57 (79.2)
Prior radiotherapy, *n* (%)	34 (70.8)	18 (75.0)	52 (72.2)

^a^Hormone receptor status: If a patient’s tumor was ER positive or PR positive then the tumor was considered hormone-receptor positive; if ER negative and PR negative, then hormone-receptor negative

ECOG, Eastern Cooperative Oncology Group; ER, estrogen receptor; IV, intravenous; PR, progesterone receptor

### Efficacy

**Fig. 2:**
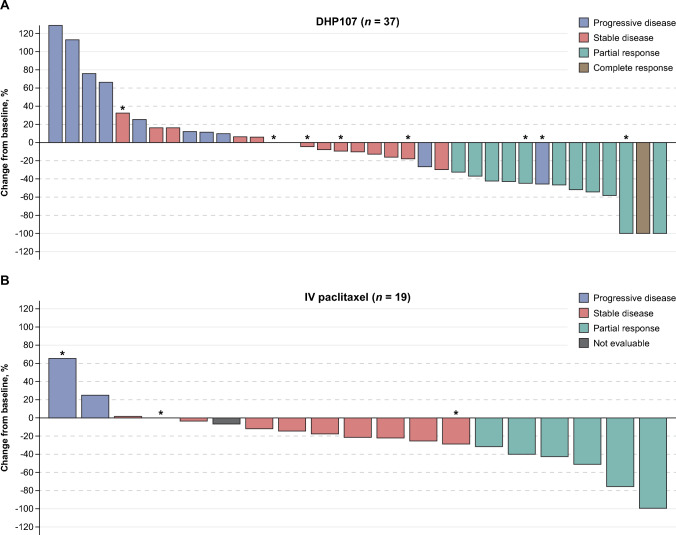
Best percent change from baseline in tumor volume (full analysis set; *n* = 69^a^). Asterisks represent patients with triple-negative breast cancer. ^a^Thirteen patients are not included in this analysis: 12 had no tumor assessment data after baseline and one had an assessment that was excluded as it was performed after a subsequent anticancer treatment IV, intravenous

Efficacy data are summarized in Table 2 and Fig. 2. There was one complete response and 11 partial responses in the DHP107 group, for an ORR of 25.0% (90% CI 15.1–37.3); there were six partial responses in the IV paclitaxel group, for an ORR of 28.6% (90% CI 13.2–48.7; *p* = 0.7559; Pearson’s chi-square test). A statistically significant difference favoring IV paclitaxel was observed in DCR, which was 54.2% (90% CI 41.4–66.6) for DHP107 and 76.2% (95% CI 56.3–90.1) for IV paclitaxel (*p* = 0.0846; Pearson’s chi-square test; statistically significant at 10% two-sided significance level). The median DoR was 7.4 months for DHP107 and 7.5 months for IV paclitaxel (*p* = 0.6095; stratified log-rank test).

**Table 2:** Efficacy data (full analysis set; *n* = 69)

	DHP107 (*n* = 48)	IV paclitaxel (*n* = 21)	*p*-value
Objective response rate, *n* (%)	12 (25.0)	6 (28.6)	0.7559^a^
[90% CI]	[15.1–37.3]	[13.2–48.7]	
Best overall response, *n* (%)			
Complete response	1 (2.1)	0	
Partial response	11 (22.9)	6 (28.6)	
Stable disease	14 (29.2)	10 (47.6)	
Progressive disease	12 (25.0)	2 (9.5)	
Not evaluable	10 (20.8)	3 (14.3)	
Disease control rate, *n* (%)	26 (54.2)	16 (76.2)	0.0846^a^,*
Median progression-free survival, months	5.5	4.7	0.8018^b^
[90% CI]	[3.5–7.3]	[3.3–8.6]	
Median overall survival, months	17.1	13.2	0.7629^b^
[90% CI]	[13.1–19.1]	[9.3–27.6]	

^a^Pearson’s chi-square test or Fisher’s exact test

^b^Unstratified log-rank test

^*^Statistically significant at 10% two-sided significance level

CI, confidence interval; IV, intravenous

**Fig. 3:**
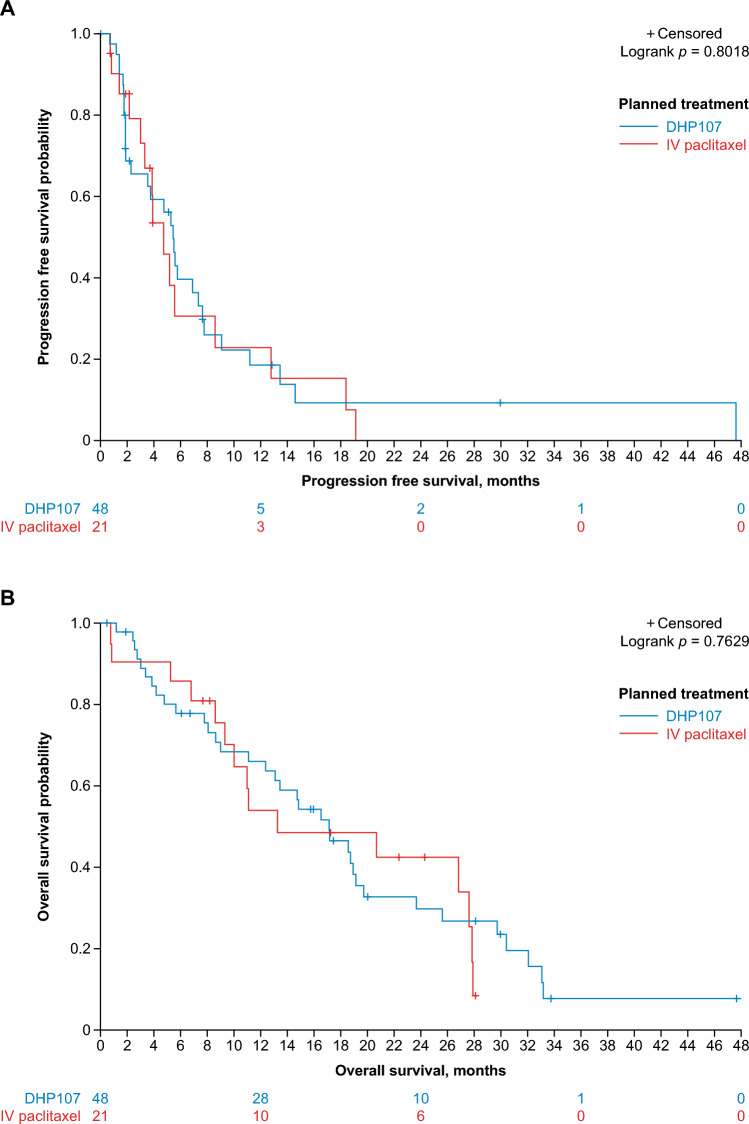
Kaplan–Meier plots of **A** progression-free survival and **B** overall survival (full analysis set; *n* = 69) IV, intravenous

The median PFS was 5.5 months for DHP107 and 4.7 months for IV paclitaxel (*p* = 0.8018; unstratified log-rank test) and median OS was 17.1 and 13.2 months, respectively (*p* = 0.7629; unstratified log-rank test) (Fig. 3). The median TTF was 2.2 months for DHP107 and 3.7 months for IV paclitaxel (*p* = 0.7222; unstratified log-rank test). Forest plots for ORR, PFS, and OS are shown in Fig. S1.

### Safety

In total, patients in the DHP107 group received a median of 2.5 (range 1–50) cycles of treatment and those in the IV paclitaxel group received a median of 4 (range 1–21) cycles of treatment. Adverse drug reactions (ADRs) for which a causal relationship to the study drug could not be ruled out occurred in 47 of 48 patients in the DHP107 group (97.9%) and 19 of 21 patients in the IV paclitaxel group (90.5%). Serious TEAEs occurred in 10 patients (20.8%) in the DHP107 group and six patients (28.6%) in the IV paclitaxel group. Serious ADRs occurred in four patients (8.3%) in the DHP107 group and one patient (4.8%) in the IV paclitaxel group. TEAEs resulted in treatment interruption, dose reduction, or discontinuation in 41.7%, 8.3%, and 27.1% of patients in the DHP107 group and in 57.1%, 9.5%, and 28.6% of those in the IV paclitaxel group, respectively. One patient (2.1%) had a TEAE leading to death in the DHP107 group (cardio-respiratory arrest; not related to DHP107) and two patients (9.5%) in the IV paclitaxel group had a TEAE leading to death (one cardio-respiratory arrest and one septic shock; not related to IV paclitaxel). There was no statistically significant difference between the groups in TEAEs leading to study drug interruption, study drug reduction, study drug discontinuation, or death.

The most common all-grade AEs in the DHP107 group were diarrhea (*n* = 33; 68.8%), nausea (*n* = 31; 64.6%), and fatigue (*n* = 25; 52.1%); the most common all-grade AEs in the IV paclitaxel group were fatigue (*n* = 10; 47.6%), peripheral neuropathy (*n* = 9; 42.9%), and alopecia (*n* = 9; 42.9%), although alopecia rates were similar between the two arms (Table 3). Febrile neutropenia was not reported. Diarrhea, nausea, decreased neutrophil count, and headache were statistically significantly more frequent with DHP107 (all *p* < 0.1 vs IV paclitaxel); dyspnea, anemia, peripheral neuropathy, and infusion-related reaction were statistically significantly more common with IV paclitaxel (all *p* < 0.1 vs DHP107). Vomiting occurred in numerically more patients in the DHP107 arm (*n* = 22; 45.8%) than in the IV paclitaxel arm (*n* = 5; 23.8%), a difference that was not statistically significant (*p* = 0.1107). The most common grade 3 or higher AE was decreased neutrophil count (DHP107, 31.3% vs IV paclitaxel, 9.5%; *p* = 0.0709). Serious AEs occurred in 10 patients (20.8%) in the DHP107 group and six patients (28.6%) in the IV paclitaxel group (*p* = 0.5417). AEs resulting in study drug discontinuation occurred in 13 patients in the DHP107 group (27.1%) and six patients in the IV paclitaxel group (28.6%; *p* = 1.00).

**Table 3:** Treatment-emergent adverse events occurring in > 20% of patients (safety set; *n* = 69)

	DHP107 (*n* = 48)		IV paclitaxel (*n* = 21)		*p*-value	
Event, *n* (%)	All grades	Grade ≥ 3	All grades	Grade ≥ 3	All grades	Grade ≥ 3
Diarrhea	33 (68.8)	5 (10.4)	6 (28.6)	1 (4.8)	0.0033*	0.6591
Nausea	31 (64.6)	0	8 (38.1)	1 (4.8)	0.0640*	0.3043
Fatigue	25 (52.1)	4 (8.3)	10 (47.6)	0	0.7972	0.3058
Vomiting	22 (45.8)	0	5 (23.8)	0	0.1107	NA
Alopecia	20 (41.7)	0	9 (42.9)	0	1.0000	NA
Neutrophil count decreased	19 (39.6)	15 (31.3)	2 (9.5)	2 (9.5)	0.0211*	0.0709*
Headache	17 (35.4)	0	3 (14.3)	0	0.0905*	NA
Abdominal pain	11 (22.9)	1 (2.1)	2 (9.5)	0	0.3166	1.0000
Decreased appetite	11 (22.9)	0	2 (9.5)	0	0.3166	NA
White blood cell count decreased	10 (20.8)	6 (12.5)	2 (9.5)	1 (4.8)	0.3208	0.4268
Back pain	9 (18.8)	0	5 (23.8)	0	0.7468	NA
Cough	9 (18.8)	0	5 (23.8)	0	0.7468	NA
Arthralgia	7 (14.6)	0	5 (23.8)	0	0.4908	NA
Dyspnea	7 (14.6)	0	8 (38.1)	1 (4.8)	0.0538*	0.3043
Anemia	6 (12.5)	2 (4.2)	9 (42.9)	2 (9.5)	0.0095*	0.5799
Constipation	6 (12.5)	0	5 (23.8)	0	0.2901	NA
Neuropathy peripheral	6 (12.5)	0	9 (42.9)	0	0.0095*	NA
Infusion-related reaction	0	0	6 (28.6)	1 (4.8)	0.0005*	0.3043

IV, intravenous; NA, not applicable

^*^Statistically significant at 10% two-sided significance level

Although permitted in the protocol, granulocyte-colony stimulation factor was not administered during study treatment. Twenty-one patients received prophylactic antiemetic medication, most commonly ondansetron (*n* = 20; 27.8%). The use of ondansetron was numerically more common in the DHP107 group (*n* = 15; 31.3%) than in the IV paclitaxel group (*n* = 5; 20.8%), which may have contributed to the greater incidence of headache.

### Pharmacokinetics

Thirteen patients who received DHP107 in OPERA were enrolled in the PK substudy; all were White and female. One patient withdrew from the PK study because of nausea and was replaced with another patient. The demographic characteristics of patients in the PK group are shown in Table S1 in the Supplementary material.

DHP107 was rapidly absorbed after oral administration, with a median T_max_ of 2.17 h (range 1.92–4.08). The mean paclitaxel concentration–time profiles are shown in Fig. S2 and overlaid individual curves are shown in Fig. S3 in the Supplementary material. After T_max_, DHP107 was rapidly eliminated, with a mean terminal elimination half-life of 3.44 h. C_max_ and AUC_last_ (coefficients of variation) were 330 ng/mL (31.1%) and 1233 ng∙h/mL (30.3%), respectively. Summary statistics for PK parameters are shown in Table S2 in the Supplementary material; PK parameters for Asian patients who were treated with DHP107 in the phase 1 study by Hong and colleagues [13] are also shown in Table S2 in the Supplementary material.

### Health-related quality of life

Baseline HRQoL data were available for 48 patients in the DHP107 group and 21 patients in the IV paclitaxel group. Global Health Status scores over the course of the study are shown in Fig. S4 in the Supplementary material. Scores were largely similar throughout the study. The number of patients with data was limited in both groups by cycle 9.

## Discussion

Treatment for patients with HER2-negative MBC generally includes chemotherapy for TNBC, and endocrine therapy with targeted agents followed by chemotherapy for patients with hormone receptor-positive MBC. A significant limitation of sequential therapy is the limited availability of oral chemotherapy options, including only capecitabine and, in some countries, vinorelbine. Taxanes remain a highly effective treatment option, limited by cumulative risk of peripheral neuropathy, which can have a significant impact on the patient’s quality of life.

IV paclitaxel is hydrophobic and hence must be given in combination with Cremophor EL, a formulation agent that is associated with a range of biological effects, including neurotoxicity and hypersensitivity [4]. Peripheral neuropathy, the primary neurotoxicity associated with IV paclitaxel, is cumulative, variable among different patients with known but complex pharmacogenomic factors, and persists for months and even years after discontinuing treatment [14]. Hypersensitivity reactions are uncommon, but occur in a subset of patients receiving Cremophor EL. Although these reactions can be minimized using slower infusion rates and premedication with corticosteroids and histamine antagonists, they remain a significant risk [4]. Improving the tolerability of taxanes and expanding access to effective orally administered chemotherapy are ongoing unmet needs. The requirement for IV administration of chemotherapy in the advanced-stage setting poses additional issues for patients that impact qualify of life, as the frequency of administration and risk of local venous irritation often requires central venous access.

The oral paclitaxel formulation DHP107 was designed to avoid the complications associated with IV administration of paclitaxel described above. In the OPERA study, treatment with DHP107 in patients with HER2-negative recurrent or MBC showed comparable efficacy and a differential safety profile compared with IV paclitaxel. The ORR of 25% and DCR of 54% for DHP107 reported in our study are somewhat lower than those reported in the OPTIMAL phase II study [8] and the OPTIMAL phase III study [7]. This is explained by differences in patient populations, as both OPTIMAL studies included patients with no prior chemotherapy for advanced disease, whereas patients in OPERA had received up to three prior lines of chemotherapy in the metastatic setting. Similarly, a somewhat higher ORR was reported for an oral paclitaxel formulation (paclitaxel plus the P-glycoprotein inhibitor encequidar) in a phase III study comparing the oral paclitaxel formulation versus IV paclitaxel in patients with MBC [15]. In that study, patients in the oral paclitaxel + encequidar group had an ORR of 35.8%, compared with 23.4% for the IV paclitaxel group (*p* = 0.01). Once again, however, 69.2% of patients in the study overall had received no treatment for metastatic disease (69.8% in the oral paclitaxel + encequidar group and 67.9% in the IV paclitaxel group); fewer than one-third of patients had previously received a taxane (28.7% and 31.4%, respectively), most of whom had received the taxane in the adjuvant setting. The ORR in OPERA is within the range of results from prior studies of paclitaxel monotherapy in patients with previously treated MBC [15, 16]. Data from the phase III part of OPTIMAL, a noninferiority study, suggest that DHP107 is an effective and convenient alternative to IV paclitaxel in this setting. DHP107 was shown to be noninferior to IV paclitaxel, with median PFS of 10.0 versus 8.5 months, respectively (HR 0.869; 95% CI 0.707–1.068) [7]. OS was comparable in both groups of patients, with median OS of 33.0 and 32.5 months, respectively (HR 0.979; 95% CI 0.769–1.246).

The safety profile of DHP107 in patients in OPERA was consistent with that in previous studies and no new safety signals were identified [6–8, 17]. Patients in the DHP107 group had statistically significantly lower rates of all-grade peripheral neuropathy, dyspnea, anemia, and infusion reactions, but higher rates of neutropenia, diarrhea, nausea, and headache compared with those receiving IV paclitaxel. Peripheral neuropathy rates were also lower for DHP107 versus IV paclitaxel in OPTIMAL III, the study for which OPERA was designed to provide support [6, 7]. Although clearly this may vary between individual patients, neutropenia and diarrhea are generally predictable and reversible with effective prophylactic strategies compared with peripheral neuropathy and hypersensitivity reactions. Nausea was also more frequent in patients taking DHP107, and was most commonly managed with ondansetron premedication; this was not mandatory, however, and was only administered to 14.6% of patients in the DHP107 group. Headache, which was numerically more common in the DHP107 group, may have been a side effect of ondansetron premedication, use of which was more common in the DHP107 group. Rates of alopecia were lower than expected, for which there are several possible explanations. The treatment period was shorter than in other studies of patients with the same indication. The study was conducted during the COVID-19 pandemic, during which time it was difficult to run trials smoothly and treatment may not have continued for as long as it might otherwise have done. The pandemic setting also resulted in suboptimal collection of data, in particular AE data, with remote monitoring replacing on-site visits. There were no statistically significant differences between the groups in TEAEs leading to study drug interruption, study drug reduction, study drug discontinuation, or death.

The higher incidence of peripheral neuropathy in the IV paclitaxel group is consistent with results of prior studies [6, 15], and suggests that the neurotoxicity associated with Cremophor EL [4] could be largely avoided with use of DHP107. The novel taxane nab-paclitaxel, constructed with protein rather than Cremophor EL, has also been associated with markedly reduced hypersensitivity and reduced peripheral neuropathy, although there is still clearly a cumulative effect. Also, nab-paclitaxel must be given intravenously, and oral therapy is clearly seen by patients as an advantage over IV administration. The increased rate of nausea is a consideration with oral agents, as previously reported for paclitaxel + encequidar [15], and was more common with DHP107 versus IV paclitaxel, which is a low emetogenic agent. However, the use of newer antiemetics such as olanzapine [18] might further reduce this toxicity and increase the tolerability of DHP107.

PK profiles were well characterized from plasma concentrations in 13 patients up to 10 h after oral administration of DHP107. DHP107 was rapidly absorbed and eliminated, and inter-individual variability in exposure such as C_max_ and AUC_last_ was low. Compared with previous phase I PK results in South Korean patients [13], C_max_ and AUC parameters were similar after dosing with DHP107, suggesting no clinically significant differences between Asian and non-Asian patients.

Some limitations of the study should be considered. This was a small study including patients who had heterogeneous treatment histories and numbers of prior lines of therapy. A considerable number of patients in the FAS were not included in the PPS. There was no mandatory antiemetic protocol for participants in the DHP107 group, possibly resulting in suboptimal uptake of therapy in this group. The study was conducted during the COVID-19 pandemic, resulting in significant challenges that have been discussed above.

In summary, DHP107, an oral paclitaxel, was a generally tolerable and feasible treatment for patients with recurrent or metastatic HER2-negative breast cancer, with similar efficacy and safety to IV paclitaxel in this setting.

## Supplementary Information

Below is the link to the electronic supplementary material.

Supplementary file1 (DOCX 902 kb)

## Data Availability

Daehwa Pharmaceutical CO., LTD’s data sharing policy is compliant with ICMJE guidelines. Daehwa Pharmaceutical CO., LTD. will consider reasonable requests for clinical data from qualified researchers. Data may be shared, at Daehwa Pharmaceutical CO., LTD’s sole discretion, with external researchers whose proposed use of the data has been approved. Complete deidentified data will be eligible for sharing 2 years after completion of the relevant study. Before release of any data, the recipient must enter into a data sharing agreement with Daehwa Pharmaceutical CO., LTD., after which the deidentified data sets can be accessed within a secured portal.
