# Supplementary material for: OPERA: a phase II study of DHP107 (oral paclitaxel) versus intravenous paclitaxel in patients with HER2-negative recurrent or metastatic breast cancer
Source: Breast Cancer Res Treat. 2026 Mar 30;217(1):5. doi: 10.1007/s10549-026-07944-2 (PMC13035570; doi:10.1007/s10549-026-07944-2)
Supplement: Supplementary file 1 — Supplementary file1 (DOCX 902 kb) [file 10549_2026_7944_MOESM1_ESM.docx]

# OPERA: a phase II study of DHP107 (oral paclitaxel) versus intravenous paclitaxel in patients with HER2-negative recurrent or metastatic breast cancer

# Supplementary information

## Table S1 Patient demographics (PK group; *n* = 13)

| Study site | Patient no. | Age (years) | Dose (mg) |
| --- | --- | --- | --- |
| MGH | 1 | 51 | 350 |
| SLCI | 2 | 57 | 350 |
|  | 3 | 66 | 350 |
|  | 4 | 48 | 300 |
|  | 5 | 79 | 350 |
| KUMC | 6 | 55 | 400 |
|  | 7 | 61 | 400 |
|  | 8* | 69 | 300 |
|  | 9 | 82 | 350 |
|  | 10 | 53 | 300 |
|  | 11 | 59 | 300 |
|  | 12 | 63 | 250 |
|  | 13 | 46 | 350 |
| Mean |  | 61.9 |  |
| SD |  | 10.6 |  |
| Range |  | 48−82 |  |

*One patient terminated early and did not complete the PK sampling on day 8 pre-dose. An additional patient was therefore enrolled into the PK group according to the study protocol

KUMC, University of Kansas Medical Center (site 06); MGH, Massachusetts General Hospital (site 03); PK, pharmacokinetic; SD, standard deviation; SLCI, Saint Luke’s Cancer Institute (site 05)

## Table S2 Summary statistics for pharmacokinetic parameters for DHP107 (*n* = 13) and historical pharmacokinetic parameters for IV paclitaxel (*n* = 3) [13]

| Statistic | T_max_  (h) | T_½_ (h) | C_max_ (ng/mL) | AUC_last_ (ng·h/mL) | AUC_inf_ (ng·h/mL) |
| --- | --- | --- | --- | --- | --- |
| **DHP107** |  |  |  |  |  |
| *n* | 13 | 11 | 13 | 13 | 11 |
| Mean (SD) | – | 3.44 (0.93) | 330 (103) | 1233 (374) | 1462 (411) |
| CV% | – | 27.1 | 31.1 | 30.3 | 28.1 |
| Median | 2.17 | – | – | – | – |
| [Min; max] | [1.92; 4.08] | – | – | – | – |
| **IV paclitaxel** |  |  |  |  |  |
| Mean (SD) | 4.0 (3.0–approx. 6.0) | 24.2 (7.6) | 235.0 (103.2) | 1348.1 (265.0) | NR |

AUC_inf_, area under the curve from the first time point extrapolated to infinity; AUC_last_, area under the curve to the last quantifiable time point; C_max_, maximum concentration; CV, coefficient of variation; IV, intravenous; NR, not reported; SD, standard deviation; T_½_, half-life; T_max_, time to peak drug concentration

**Fig. S1** Forest plot for (A) objective response rate, (B) progression-free survival, and (C) overall survival

A


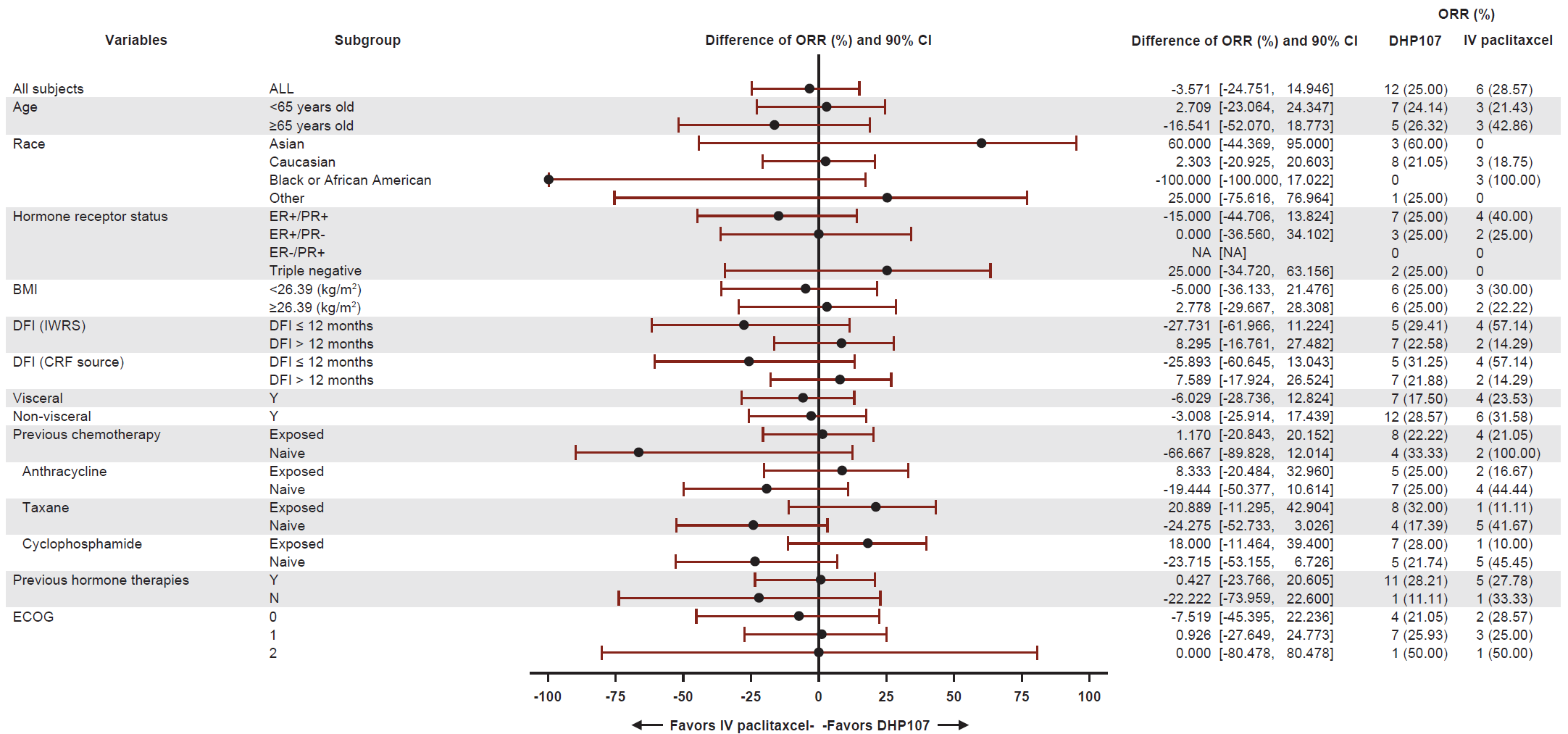


B


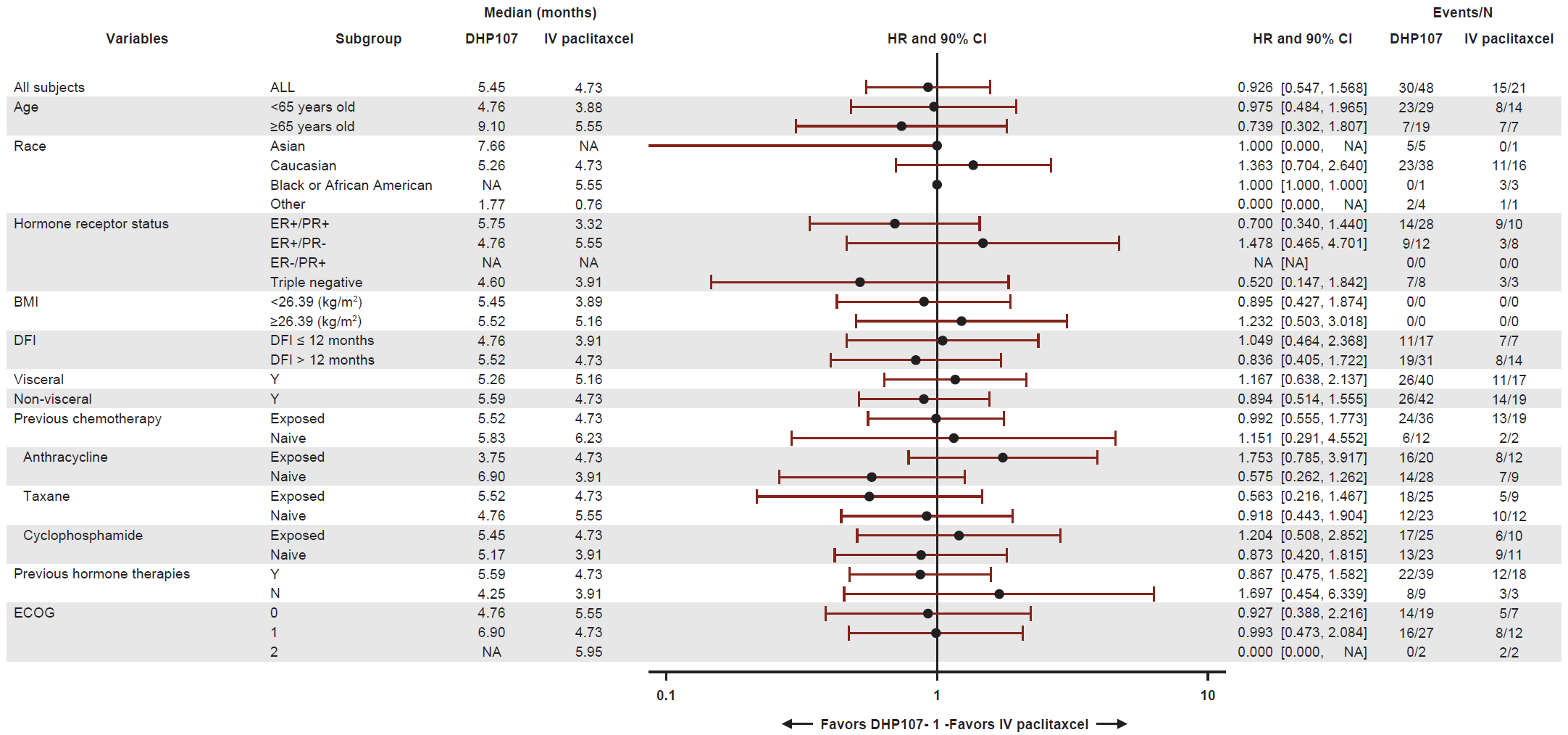


C


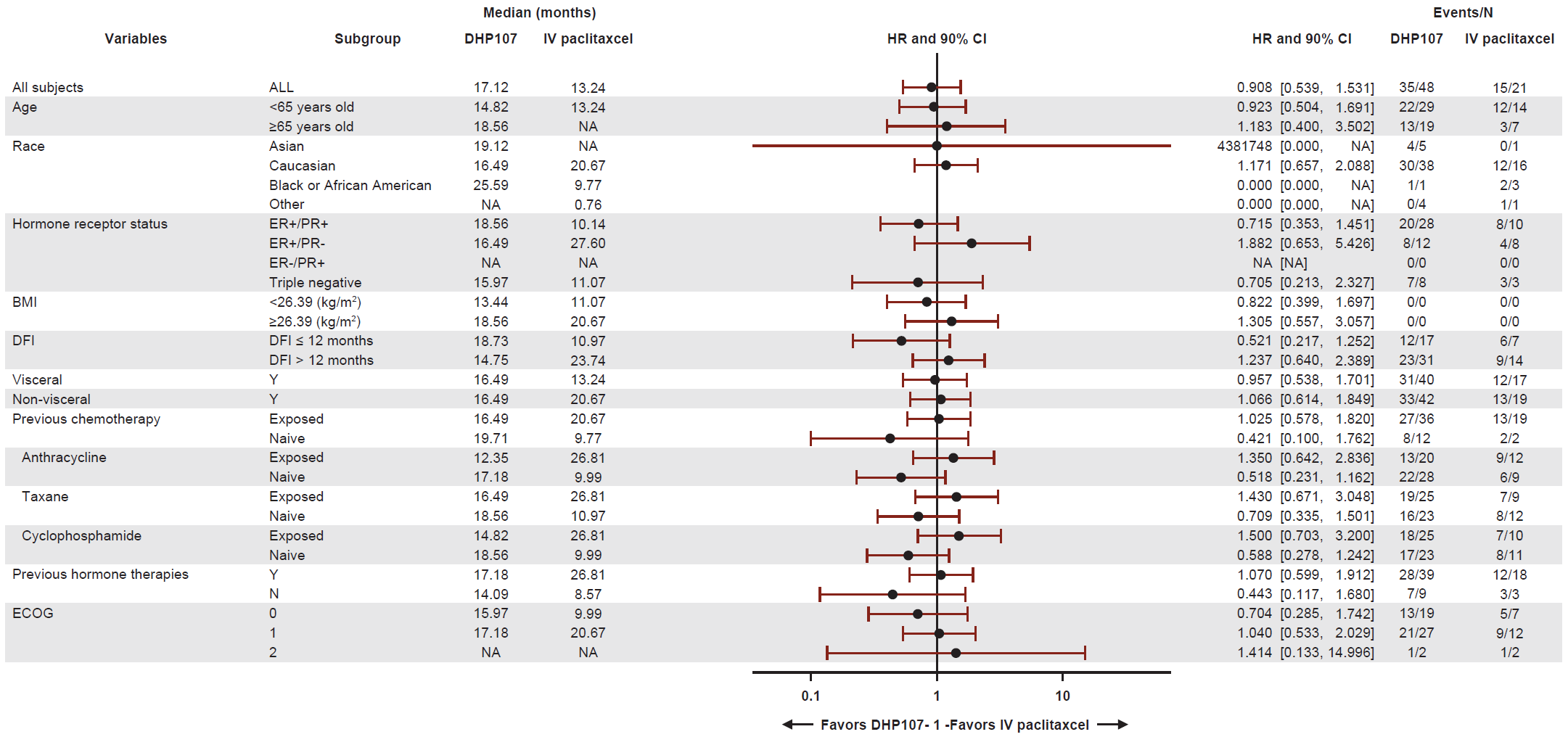


BMI, body mass index; CI, confidence interval; CRF, case report form; DFI, disease-free interval; ECOG, Eastern Cooperative Oncology Group; ER, estrogen receptor; HR, hazard ratio; IV, intravenous; N, no; NA, not applicable; ORR, objective response rate; PR, progesterone receptor; IWRS, interactive web response system; Y, yes

**Fig. S2** Mean ± standard deviation paclitaxel concentration–time profiles for patients treated with DHP107 in the OPERA pharmacokinetics substudy: (A) linear and (B) semi-log data


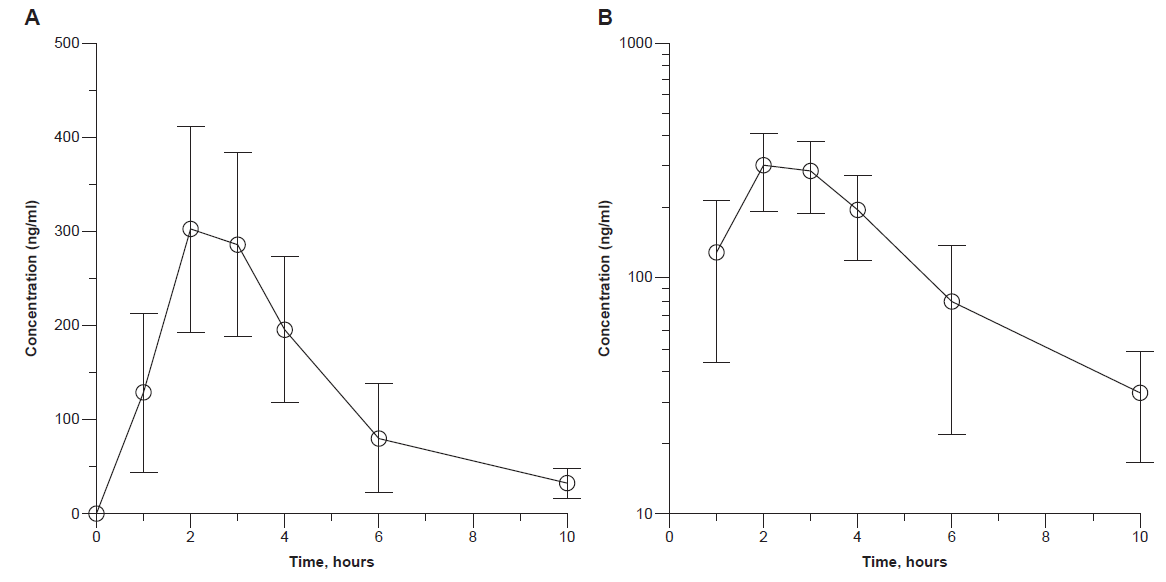


**Fig. S3** Individual paclitaxel concentration–time profiles for patients treated with DHP107 in the OPERA pharmacokinetics substudy: (A) linear and (B) semi-log data


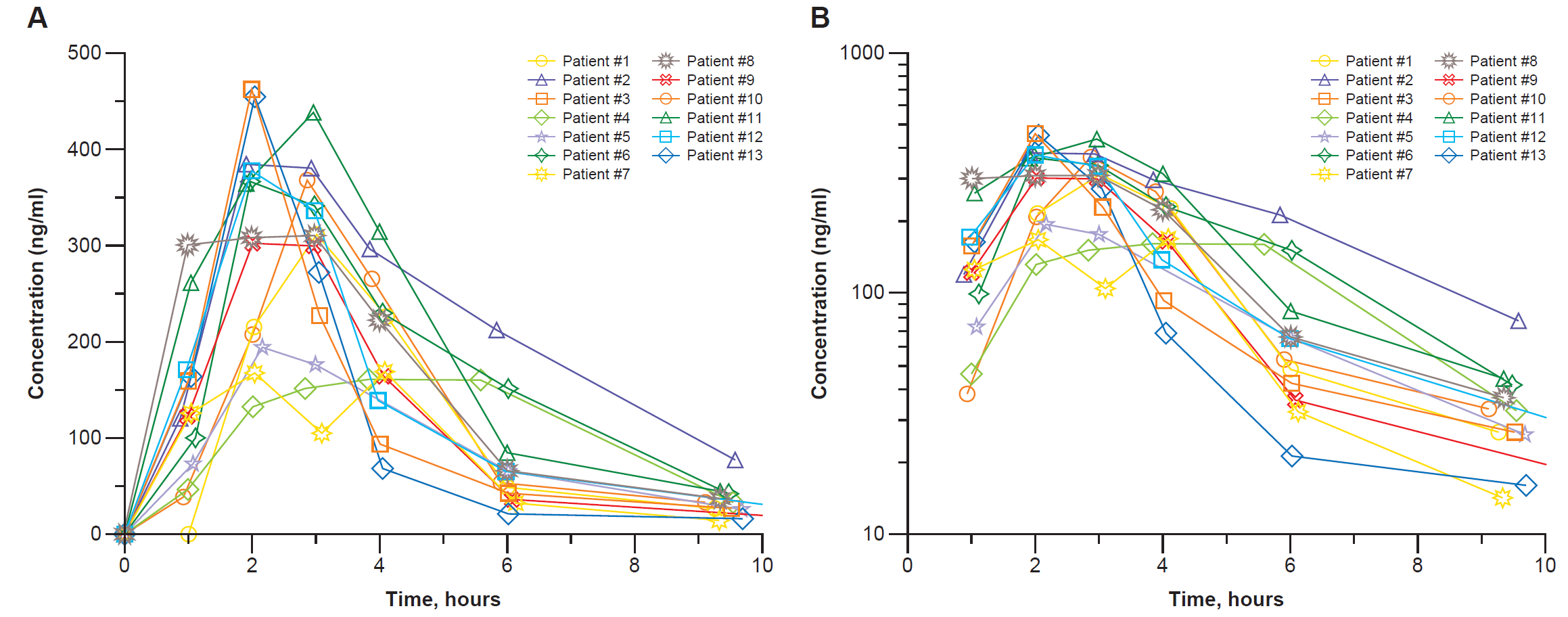


**Fig. S4.** EORTC QLQ-C30 Global Health Status scale scores^a^


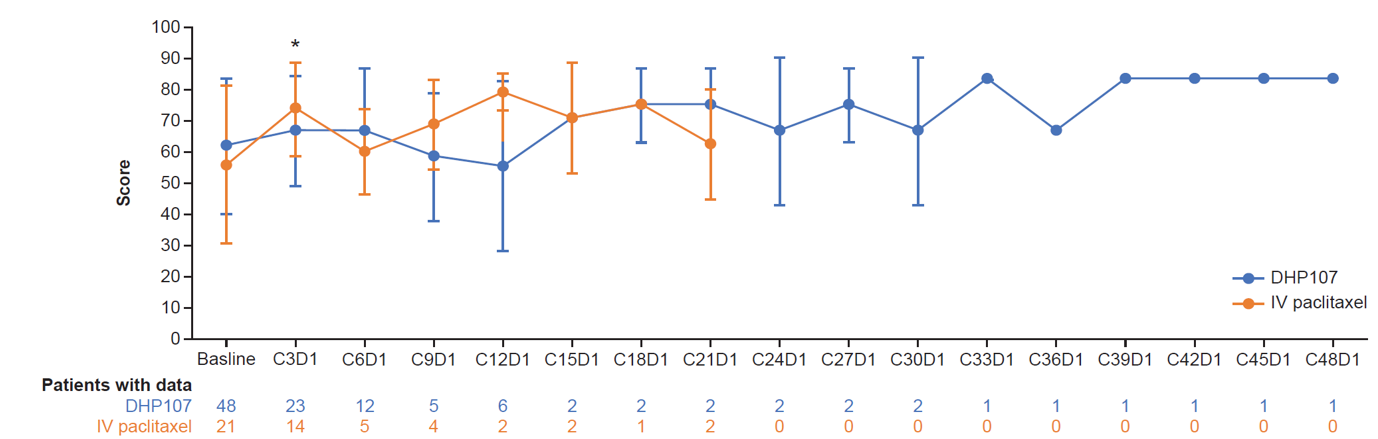


^a^Scores range from 0–100, with higher scores representing better quality of life.

*p<0.05. Statistically significant at 10% two-sided significance level.

EORTC, European Organisation for Research and Treatment of Cancer; IV, intravenous
